# Sphingosine-1-Phosphate Treatment Can Ameliorate Microvascular Leakage Caused by Combined Alcohol Intoxication and Hemorrhagic Shock

**DOI:** 10.1038/s41598-017-04157-y

**Published:** 2017-06-22

**Authors:** Travis M. Doggett, Natascha G. Alves, Sarah Y. Yuan, Jerome W. Breslin

**Affiliations:** Department of Molecular Pharmacology and Physiology, Morsani College of Medicine, University of South Florida, Tampa, FL USA

## Abstract

Fluid resuscitation following hemorrhagic shock is often problematic, with development of prolonged hypotension and edema. In addition, many trauma patients are also intoxicated, which generally worsens outcomes. We directly investigated how alcohol intoxication impacts hemorrhagic shock and resuscitation-induced microvascular leakage using a rat model with intravital microscopic imaging. We also tested the hypothesis that an endothelial barrier-protective bioactive lipid, sphingosine-1-phosphate (S1P), could ameliorate the microvascular leakage following alcohol intoxication plus hemorrhagic shock and resuscitation. Our results show that alcohol intoxication exacerbated hemorrhagic shock and resuscitation-induced hypotension and microvascular leakage. We next found that S1P effectively could reverse alcohol-induced endothelial barrier dysfunction using both cultured endothelial cell monolayer and *in vivo* models. Lastly, we observed that S1P administration ameliorated hypotension and microvascular leakage following combined alcohol intoxication and hemorrhagic shock, in a dose-related manner. These findings suggest the viability of using agonists that can improve microvascular barrier function to ameliorate trauma-induced hypotension, offering a novel therapeutic opportunity for potentially improving clinical outcomes in patients with multi-hit injuries.

## Introduction

A major clinical challenge with the resuscitation of trauma patients who have undergone severe loss of blood is the systemic leakage of plasma from the microcirculation. The elevated microvascular permeability is characterized by changes in the junctions between cells that form the endothelial barrier^1, 2^. Local inflammatory mediators are known to elicit elevated microvascular permeability, as are second-hits that often present with hemorrhagic shock injury in the clinic, such as alcohol intoxication, producing increased injury severity^3–6^. To date, strategies to restore mean normal plasma volume in hemorrhagic shock patients while preventing additional microvascular leakage have not been successful^7, 8^.

Binge drinking in the United States produces an estimated $223.5 billion cost burden to society^9^ because of its role in motor-vehicle accidents, homicides, suicides, and interpersonal violence^5, 10^. Previous studies have shown that up to 80% of alcohol-intoxicated trauma patients present with hemorrhagic shock, and are significantly more hypotensive upon arrival to the emergency department, requiring significantly greater volumes of resuscitative fluids, and having poorer outcomes^11–13^. To date, research has been focused on neuroendocrine and metabolic mechanisms that regulate vascular tone, or on hypertonic fluids to restrict plasma leakage from the central circulation^14–17^. However, these approaches have produced underwhelming results in the clinic^7, 8^. We recently discovered that acute alcohol intoxication produces microvascular plasma protein leakage *in vivo* ^18^. This finding led us to hypothesize that the severe hypotension following combined alcohol intoxication and hemorrhagic shock is due in large part to microvascular leakage of plasma, and that a therapeutic strategy to enhance endothelial barrier integrity would prevent loss of central fluids and thus improve overall hemodynamic stability.

## Results

### Alcohol Intoxication Worsens Hemorrhagic Shock and Resuscitation (HSR)-Induced Microvascular Leakage

**Figure 1:**
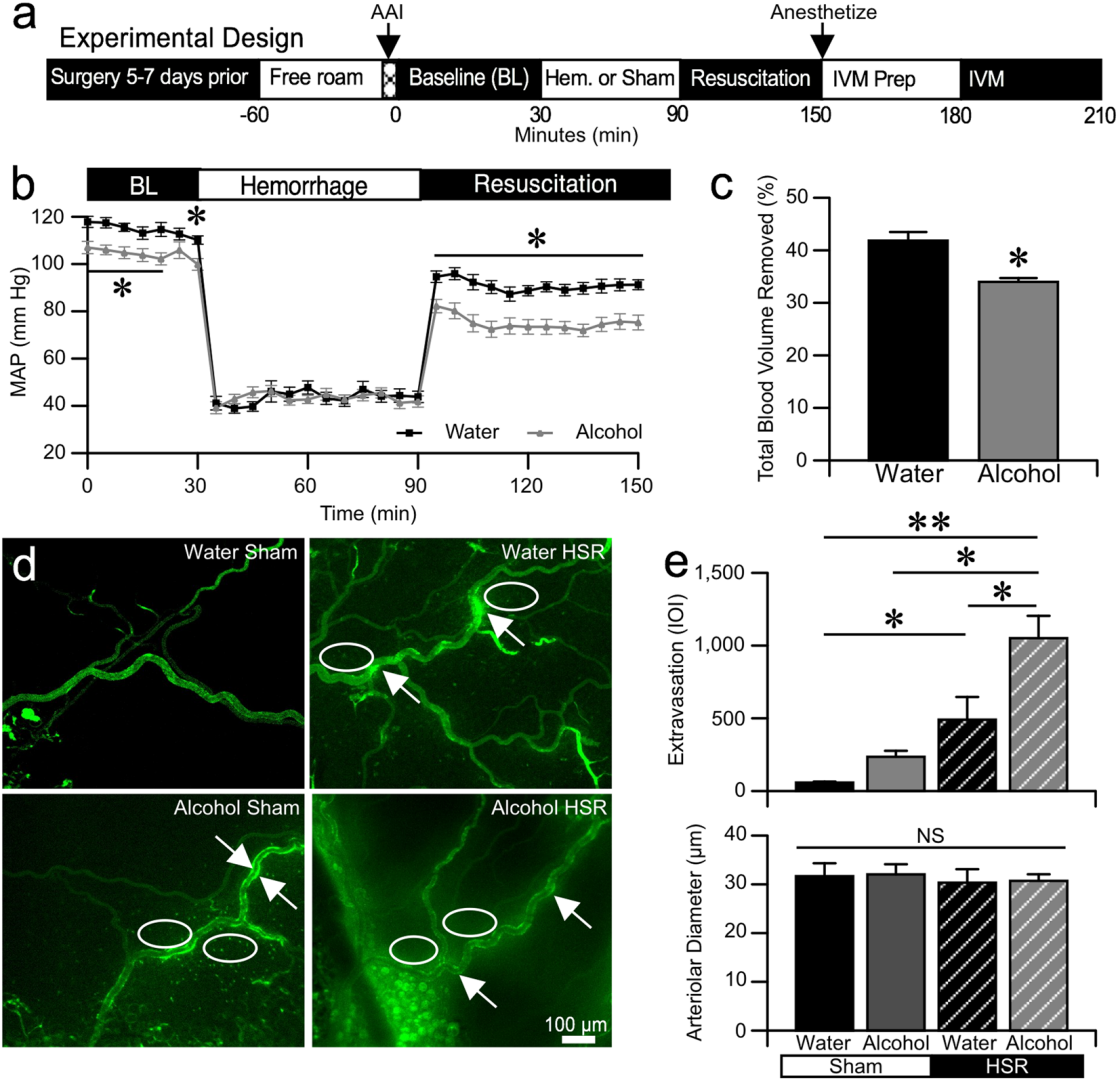
Acute alcohol intoxication worsens HSR-induced microvascular hyperpermeability. (**a**) Experimental design. Gastric and vascular catheter implantation surgery was performed on male Sprague-Dawley rats, followed by 5–7 days for recovery. Acute alcohol intoxication (Alcohol) consisted of a 2.5 g/kg bolus via the intragastric catheter. The bolus was delivered in the 3–5 min. period just prior to the start of the 30-min baseline period. Hemorrhage (Hem.) was a fixed-pressure hemorrhage to 40–60 mm Hg. Resuscitation (Res.) consisted of warm LR as a 40% total blood volume removed (TBR) bolus and 2X TBR infusion for 1 h. Following resuscitation, the rats were anesthetized, prepped for intravital microscopy, and the mesenteric microcirculation imaged over a 30-min period. (**b**) Time-course changes in mean MAP of control/water (black squares, n = 5) and Alcohol (grey triangles, n = 7) rats during fixed-pressure hemorrhagic shock and resuscitation. Values are means ± SEM. *p < 0.05 between groups at the time points indicated (repeated measures ANOVA and Fisher’s LSD test). (**c**) Percentage of total blood volume removed during the fixed-pressure phase of the HSR protocol. *p < 0.05 between groups (Student t-test). (**d**) Representative fluorescent images of the mesenteric microcirculation from the Water-Sham (n = 6), Alcohol-Sham (n = 7), Water-HSR (n = 5), and Alcohol-HSR (n = 7) groups. The ovals highlight areas of FITC-Albumin leakage into the interstitium. The arrowheads indicate areas of FITC-Albumin accumulation resulting in hotspots of fluorescence within just outside the vessel walls. (**e**) Comparison of FITC-albumin fluorescence in the extravascular space, measured by integrated optical intensity (IOI), and the mean arteriolar diameter between the aforementioned groups. Values are means ± SEM. *p < 0.05 and **p < 0.01 between groups (Two-way ANOVA and Tukey test).

We found that combined alcohol intoxication and hemorrhagic shock injury increased microvascular leakage compared to either of these insults alone (Fig. 1). Employing the illustrated experimental design (Fig. 1a), male Sprague-Dawley rats were administered alcohol or isovolumic water intragastrically, and subjected to either a 1-h fixed-pressure hemorrhage and resuscitation or a time-matched control period with no interventions, during which blood pressure was monitored^16, 17^. For rats receiving alcohol administration, the blood alcohol concentrations (BAC), measured at the time blood was drawn to produce hemorrhage, was 128.3 ± 8.3 mg/dL. In the hemorrhagic shock and resuscitation (HSR) groups, intoxicated rats presented with significantly lower mean arterial blood pressure (MAP) during the 30 min following administration of alcohol and during the entire 60 min of resuscitation period, compared to controls (Fig. 1b). In addition, the volume of blood removed required to achieve the fixed-pressure hemorrhage was significantly less in the alcohol group compared to the water-treated group (Fig. 1c). Following completion of hemorrhagic shock and fluid resuscitation (HSR) protocol, extravasation of FITC-albumin from the mesenteric microcirculation was assessed in the mesentery by intravital microscopy in the four treatment groups: Water-Sham, Alcohol-Sham, Water-HSR, and Alcohol-HSR (Fig. 1d). In the Water-Sham group, the interstitium surrounding microvessels had very low fluorescence intensity, whereas both the Alcohol-Sham and Water-HSR groups, there was a marked elevation in fluorescence intensity in the interstitial spaces around microvessels (white ovals) and “hot spots” of intensity on or near the vessel walls (white arrows). Alcohol-HSR animals displayed even greater interstitial fluorescence intensity and hot spots near the vessel walls. The quantified data show that the Alcohol-Sham and Water-HSR groups had significantly greater integrated optical intensity (IOI) in the extravascular areas compared to Water-Sham (p < 0.05), and an even further elevated IOI with the Alcohol-HSR group (Fig. 1e). Average arteriolar diameter, a key determinant for local tissue perfusion, was similar between all groups tested, suggesting that the microvascular leakage is due to increased permeability rather than increased filtration^19^. These data suggest that acute alcohol intoxication worsens hemorrhagic shock-induced microvascular hyperpermeability.

### S1P Rapidly Reverses Alcohol-Induced Endothelial Barrier Dysfunction

**Figure 2:**
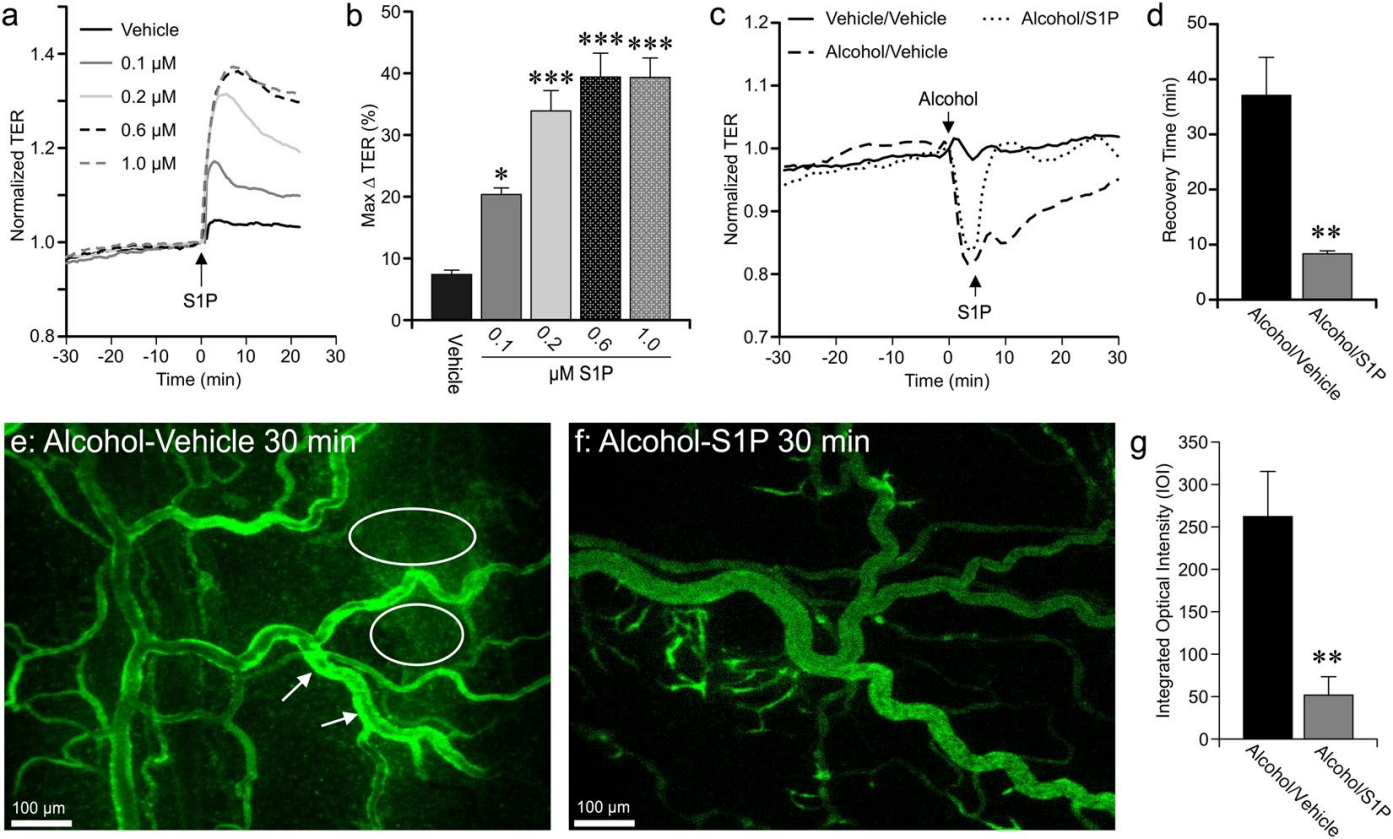
S1P significantly attenuates alcohol-induced microvascular hyperpermeability *in vivo*. (**a**) Time-course of changes in mean TER of HUVEC monolayers pretreated with 0.1, 0.2, 0.6, or 1 µM S1P or vehicle (PBS). (**b**) The mean maximum change in TER following addition of S1P, compared to the time point immediately before S1P was added. *p < 0.05 and ***p < 0.001 vs. vehicle (ANOVA and Dunnett test). For all groups, n = 8 cell monolayers studied. (**c**) Time course of changes in HUVEC monolayer TER in response to 50 mM alcohol, followed by addition of 1 µM S1P or vehicle (PBS) 5 min later. (**d**) Mean recovery time to re-achieve baseline TER, starting from the time point immediately preceding alcohol was added. **p < 0.01 vs. alcohol/vehicle group (Student t-test). Values are means ± SEM. For all cell monolayer groups, n = 8. (**e**) Representative fluorescent image showing extravasation of FITC-albumin from the mesenteric microcirculation from an Alcohol-Sham rat, 30 min. after the initial infusion of FITC-albumin (approximately 1.5 h after intragastric alcohol infusion). White ovals highlight areas with elevated FITC-Albumin fluorescence in the interstitium. White arrows indicate “hotspots” of FITC-Albumin accumulation within and just outside the vessel walls. (**f**) Representative image corresponding to a time-matched rat that received a total of 0.1 mg/kg S1P during the FITC-albumin infusion. (**g**) Mean extravasation of FITC-Albumin quantified as IOI in the extravascular space. **p < 0.01 Alcohol-S1P vs. Alcohol-Vehicle (Student t-test). Values are means ± SEM. For both treatment groups, n = 8 rats.

We set out to test possible pharmacological interventions that could be employed to restore MAP during resuscitation and at least partly attenuate the enhanced microvascular hyperpermeability caused by alcohol intoxication patients and hemorrhagic trauma. Sphingosine 1-Phosphate (S1P) is a bioactive sphingolipid produced endogenously in cell membranes, and is stored and continuously released by red blood cells, platelets, and other cell types^20–22^. Previous reports indicate that S1P can enhance barrier function in cultured endothelial cells, reduce permeability to both water and albumin in isolated rat microvessels, and reduce microvascular hyperpermeability *in vivo* in rat cremaster muscle and murine lung^20, 23–25^. We confirmed S1P’s barrier enhancing properties in cultured HUVEC using a concentrations representing the range reported in plasma^21^. All concentrations produced a significant increase in TER, with a maximal increase of 40% compared to vehicle (Fig. 2a,b). We next tested the extent to which S1P could restore alcohol-induced endothelial barrier dysfunction. Alcohol reduced TER as we previously have reported^18^, and subsequent treatment with S1P caused the TER to rapidly rise to reestablish TER to the baseline level or higher, producing a more rapid recovery time compared to the vehicle group (Fig. 2c,d). We next examined the ability of S1P to abolish or attenuate acute alcohol intoxication-induced microvascular leakage *in vivo*. Alcohol-Vehicle rats revealed marked extravasation of FITC-albumin from the microcirculation into the surrounding tissues (white ovals) as well as hotspots within and just outside of the vessel walls (white arrows) (Fig. 2e). On the other hand, rats that received 0.1 mg/kg S1P in the infusion had a marked reduction in the extravasation of FITC-albumin (Fig. 2d). The mean extravascular IOI for the rats receiving intravenous S1P was significantly lower than those rats receiving vehicle (Fig. 2g). These data demonstrate the potential of S1P in attenuating microvascular hyperpermeability *in vivo* following acute alcohol intoxication.

### Intravenous S1P Ameliorates Alcohol/HSR-Induced Microvascular Leakage and Hypotension in a Dose-Related Manner

**Figure 3:**
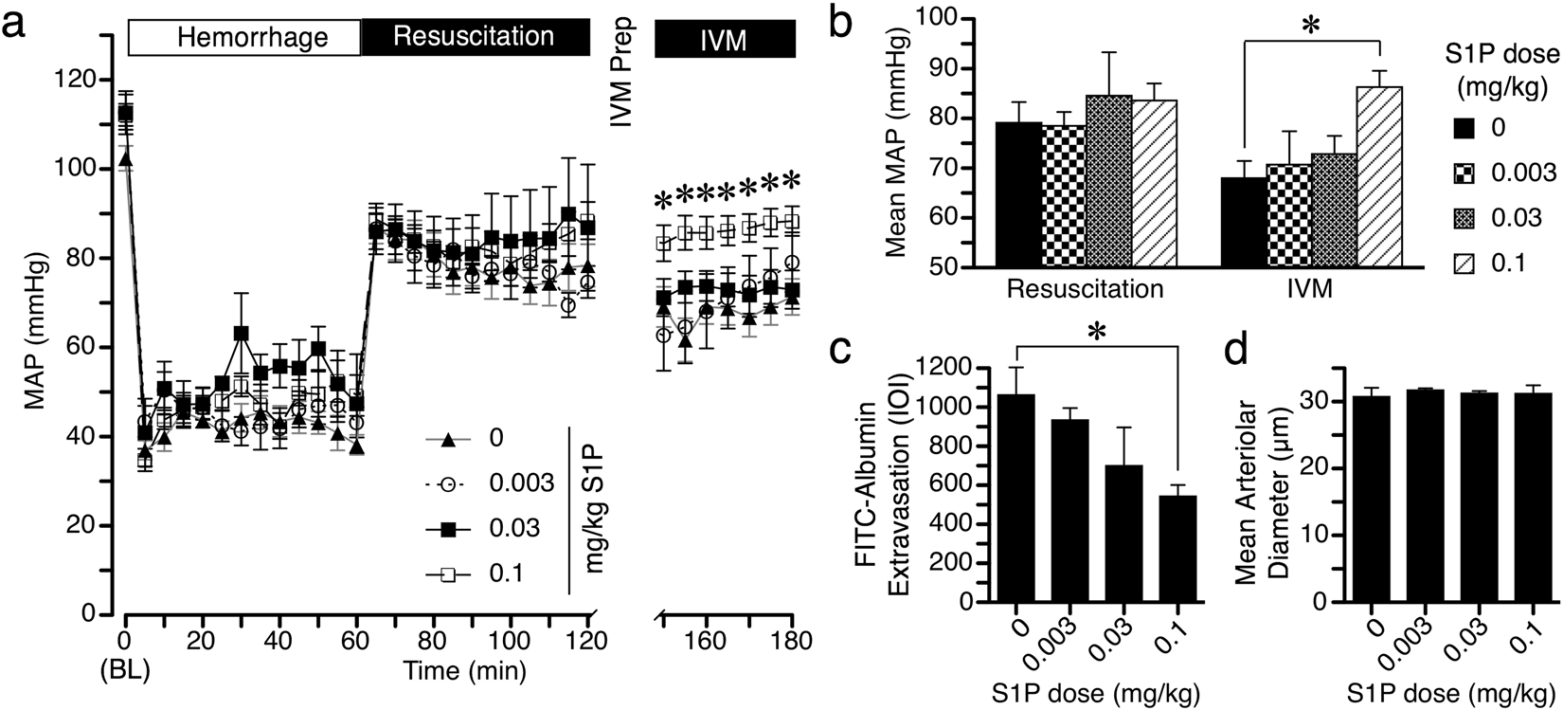
S1P administration improves hypotension and microvascular hyperpermeability caused by combined acute alcohol intoxication and hemorrhagic shock. (**a**) Time-course changes in MAP during fixed-pressure hemorrhage, resuscitation, and subsequent intravital microscopy (IVM) in rats that received S1P within LR or standard LR alone. Groups are Alcohol-HSR (filled triangles, n = 11), Alcohol-HSR + 0.003 mg/kg (open circles, n = 6), Alcohol-HSR + 0.03 mg/kg (filled squares, n = 6), and Alcohol-HSR + 0.1 mg/kg (open squares, n = 9). *p < 0.05 vs. Alcohol-HSR, same time point (repeated measures ANOVA and Dunnett test at each time point, with 0 mg/kg S1P as control). (**b**) Mean MAP of each of the previously described groups during the resuscitation or IVM phase. *p < 0.05 vs. Alcohol-HSR (ANOVA and Dunnett test). (**c**) Comparison of extravasation of FITC-Albumin, quantified as IOI in Alcohol-HSR rats that received standard LR fluid resuscitation without or with the different doses of S1P. *p < 0.05 vs. 0 mg/kg S1P (ANOVA and Dunnett test). (**d**) Arteriolar diameter in microns for each of the four groups previously described (Not significant, ANOVA). Values in each panel are means ± SEM.

Based on the data obtained *in vitro* and *in vivo*, we performed a dose-response study examining S1P’s efficacy to ameliorate combined acute alcohol intoxication and HSR-induced hypotension and elevated microvascular leakage. The same combined alcohol-intoxication and HSR protocol was performed with three doses of S1P, 0.003, 0.03, and 0.1 mg/kg, administered within the LR resuscitation fluid. During the first hour of resuscitation, 0.003 and 0.03 mg/kg S1P failed to improve MAP. However, by the end of the 1-h resuscitation phase, the rats receiving 0.1 mg/kg S1P had a significantly higher mean MAP than those not given S1P (Fig. 3a,b). We also measured MAP later, during the assessment of microvascular leakage. At this stage, the mean MAP of the 0.1 mg/kg group remained significantly higher than Alcohol-HSR for the entire 30 min intravital microscopy protocol by an average of 18 mm Hg (Fig. 3c,d). These data suggest that supplementing resuscitation fluid with 0.1 mg/kg S1P restores MAP in alcohol-intoxicated rats following HSR more effectively than LR solution alone. The hyperpermeability of the mesenteric microcirculation was not significantly reduced with the 0.003 and 0.03 mg/kg doses. However, the rats that received 0.1 mg/kg S1P demonstrated a significant reduction in the extravasation of FITC-albumin (Fig. 3e). Arteriolar diameter was measured and was found to be the same across the groups, suggesting that the reduced microvascular leakage was more likely to be due to improved endothelial barrier function rather than changes in local perfusion and filtration (Fig. 3f). In summary, these data suggest that S1P helps restore MAP in alcohol-intoxicated rats following combined alcohol intoxication and hemorrhagic shock and resuscitation by attenuating the microvascular hyperpermeability that normally accompanies hemorrhagic shock and causes loss of central fluid volume to the extravascular space.

## Discussion

The accentuated hypotensive response to blood loss during alcohol intoxication involves weakened host-defense mechanisms, decreased cellular responses to inflammatory challenge, and increased susceptibility to infections, all leading to greater morbidity and mortality^16, 17, 26, 27^. In the current study, we focused on how alcohol impacts HSR-induced microvascular permeability. The rationale for our approach was the recent discovery that alcohol intoxication alone could increase microvascular leakage in the gut mesentery^18^, which is typically susceptible to ischemic injury following HSR^7^. This mesenteric microvascular leakage occurred without changes in arteriolar or venular diameter^18^ unlike other previous work describing microvascular ruptures and microhemorrhages related to cerebral vasospasms^28, 29^. The results from the current study show for the first time that alcohol intoxication worsens HSR-induced microvascular leakage and that S1P, in a dose-related manner, reduces microvascular leakage after combined alcohol intoxication and HSR, in association with improved blood pressure during the same time frame.

A handful of other studies have also shown that S1P or its mimetics may be of therapeutic benefit for reducing microvascular permeability *in vivo*. Intra-arterial administration of either the S1P receptor-1 (S1P_1_R) activator SEW2871, or a different S1P receptor activator, FTY720, which acts upon all S1P receptors except S1P_2_R, significantly reduced histamine-induced microvascular leakage in the rat cremaster muscle^24^. In the same study, administration of S1P itself did not prevent histamine-induced microvascular leakage. However, this was attributed to activation of S1P_2_R, as co-administration of a specific S1P_2_R antagonist that is known to prevent disruption of endothelial intercellular junctions, JTE-013^30^, with S1P was effective at attenuating histamine-induced microvascular leakage^24^. In a different study utilizing LPS-induced acute lung injury in mice, administration of relatively low doses of S1P or SEW2871 significantly attenuated lung alveolar permeability^25^. Moreover, FTY720-(S)-Phosphonate, an analog that better preserves S1P receptors, reduced BAL fluid, tissue albumin, and PMN infiltration in a bleomycin-induced lung injury model^31^. Most notably, the FTY720 was shown to ameliorate systemic hypotension and Evans blue dye accumulation in the lungs in a combined trauma and HSR model in anesthetized rats^32^. Our new data using the combined alcohol intoxication and HSR model is in agreement with the data obtained from these other studies.

The molecular mechanism by which S1P enhances endothelial barrier function remains incompletely understood. S1P, through its receptors, activates second messengers that promote rapid changes in the cytoskeleton, intercellular junctions, and focal adhesions to enhance the endothelial barrier integrity^33–35^. Several investigators have shown that S1P activates multiple Rho family GTPases and elicits the phosphorylation of myosin light chains in endothelial cells^36–39^. Studies of signaling events in live endothelial cells indicate that the time course of these events is rapid, with activation of RhoA near the cell periphery and Rho kinase-mediated cell spreading^38–40^. S1P-induced preservation of the endothelial glycocalyx also likely contributes to the enhanced barrier function^41–43^.

Overall, our findings present the first demonstration that reducing microvascular leakage can effectively improve the hypotension caused by combined alcohol intoxication and hemorrhagic shock. This approach represents a significant departure from previous attempts to improve MAP by targeting neuroendocrine mechanisms to promote vasoconstriction^14–17^, and opens a new line of investigation for developing new resuscitation strategies for intoxicated trauma patients. We expect that future work targeting additional barrier protective components of plasma, investigating the long-term potential of this strategy, and examining specific S1P receptors, will produce additional advances that will produce better therapies for intoxicated trauma patients.

## Methods

### Animals

All animal protocols were performed in strict accordance with the U.S. Animal Welfare Act, U.S. Public Health Service Policy on the Humane Care and Use of Laboratory Animals, and the *Guide for the Care and Use of Laboratory Animals*. All animal experiments were performed after approval from the University of South Florida Institutional Animal Care and Use Committee (permit number IS00001315). Male Sprague-Dawley rats (Harlan) (325–350 g) were provided a standard diet (Purina Rat Chow, Ralston Purina) and water ad libitum and were housed in a controlled environment (22 °C) with controlled illumination (12 h light/12 h dark cycle) in the vivarium for a one-week acclimation period prior to surgery. Surgery was performed after the rats were anesthetized, and efforts were made to minimize pain. Following experimental protocols, all animals were humanely euthanized with Euthasol/Somnasol (87 mg/kg i.v.; Henry Schein, Dublin, OH).

### Surgical preparation

The surgical preparation for the model has been previously described in detail^14, 44^. Briefly, rats were anesthetized with isoflurane (4% induction, 2.5–1.5% maintenance), and sterile catheters were implanted in the left common carotid artery and the right external jugular vein. An additional intragastric catheter was also implanted and secured with a purse-string suture. All catheters were flushed with 0.9% sterile Sodium Chloride USP (Baxter, Deerfield, IL), thermally sealed, and routed subcutaneously using a trocar to the dorsal nape of the neck. Catheters were secured to the closed incision with suture, coiled, and wrapped with masking tape to prevent damage. Carotid catheters were used for blood pressure monitoring and blood sample withdrawal. Jugular catheters were used for administration of resuscitation fluids and fluorescent tracer for intravital microscopy. Gastric catheters were used for alcohol administration. After surgery, animals were allowed 5–7 days of recovery in the same conditions as the acclimation period before experiments. Carprofen (5 mg/kg; Putney, Portland, ME) was provided every 12 h for the first 48 h following surgery to minimize pain during recovery.

### Alcohol Administration

Alcohol (Aaper Alcohol and Chemical, Shelbyville, KY) was administered using a previously established model simulating a binge-drinking episode^45, 46^. Conscious, unrestrained rats received an intragastric bolus of alcohol (30% ethyl alcohol by volume) at 2.5 g/kg via the gastric catheter. The rats were allowed to roam in their cages for 30 minutes post alcohol administration before undergoing the HSR protocol or intravital microscopy. A time-matched control group received isovolumic administration of vehicle (Millipore-filtered water).

### Fixed-pressure HSR protocol

Conscious, unrestrained rats were subjected to fixed-pressure hemorrhage and resuscitation as previously described by Molina and colleagues^16, 17, 47^. Briefly, the rats were placed in small cages and the catheters were routed through the tops of the cages. They were allowed free roam for 60 minutes in order to provide time for the animals to acclimate and minimize stress due to handling. The carotid catheter was connected to a pressure transducer for continuous blood pressure recording using an ADInstruments PowerLab 4/35 with Quad Bridge amplifier system and LabChart software (ADInstruments, Colorado Springs, CO). Alcohol or water was administered intragastrically as described above, and baseline blood pressure measurements were recorded for 30 min. To initiate hemorrhage, arterial blood was withdrawn to achieve a fixed pressure of 40–60 mm Hg for 60 minutes. The rationale for the fixed-pressure hemorrhage model was to ensure the degree of injury was the same between alcohol and water-treated groups, as previous reports indicated that fixed-volume hemorrhage results in significantly greater injury in alcohol-treated rats^15, 48, 49^. At the end of the 60-min hemorrhage period, warm (37 °C) Lactate Ringers solution was delivered intravenously initially as a bolus with a volume of 40% of the total blood removed (TBR), followed by a 2X TBR infusion over 60 min. In some experiments, S1P (Tocris, Bristol, UK) was added to the resuscitation fluid to provide the rat a total dose consisting of 0.003, 0.03, or 0.1 mg/kg. These doses were chosen based on previously published effective doses in mice and rats^24, 25^. Following the end of the HSR protocol, the animals were immediately prepped for intravital microscopy (IVM).

### *In Vivo* Assessment of Microvascular Leakage

Microvascular leakage was assessed by intravital microscopy of the mesenteric microcirculation as previously described^50–52^. Briefly, the rats were first anesthetized with isoflurane (4% induction/1.5–2.5% maintenance). The ventral abdominal fur was shaved, and the skin cleaned using 4% Chlorhexidine gluconate solution (CareFusion, Leawood, KS), 100% ethyl alcohol, and 7.5% Povidone-iodine (Purdue Products L.P., Stamford, CT). A midline laparotomy was performed and the small intestine was exteriorized and splayed over an optical stage. The mesentery was superfused with 37 °C Ringer’s solution. Body temperature was maintained by a thermal plate placed under the rat and controlled by a thermostat connected to a rectal thermometer. Blood pressure was monitored from the carotid catheter with a pressure transducer. FITC-conjugated albumin (Sigma-Aldrich, St. Louis, MO) dissolved in Lactate Ringer’s solution was administered to the jugular vein as a bolus (1 mg/10 kg over 1–2 minutes) followed by continuous infusion (0.15 mg/kg/min) to achieve a steady-state plasma concentration. The mesenteric microcirculation was viewed with a fluorescent microscope (Nikon Eclipse E600) using a 10x objective (Nikon Instruments Inc., Natick, MA) at 488 nm excitation. Images were captured at 10 min time-points for a total of 30 min with a ThorLabs USB 2.0 Digital Camera (ThorLabs, Newton, NJ) controlled with Micromanager software^53^. The integrated optical intensity (IOI) of extravascular regions in close proximity to postcapillary venules was measured to determine the degree of extravasation of FITC-albumin^50–52^.

### Experimental Groups

The rats were randomized (simple randomization) into groups. For the initial experiments, these groups were those that received alcohol versus isovolumic water (control) prior to either hemorrhagic shock/resuscitation or sham (control), yielding four different combinations. For the second set of experiments rats were randomized into groups that received alcohol administration with or without subsequent S1P administration. For the third experiment, rats that underwent the combined alcohol treatment and hemorrhagic shock/resuscitation protocol were randomized into groups that received 0, 0.003, 0.03, and 0.1 mg/kg S1P in the resuscitation fluids. Given the nature of the protocol, it was impossible to blind the investigator to the treatments.

### Endothelial Cell Culture

Pooled human umbilical vein endothelial cells (HUVEC) were obtained from Lonza (Basel, Switzerland) and grown in endothelial growth medium (EGM2MV; Lonza) on 1.5% porcine gelatin matrix (Sigma-Aldrich, St. Louis, MO) in a 37 °C, 5% CO_2_ incubator. EGM2MV was developed as a low-serum growth media for endothelial cells, containing 5% fetal bovine serum (FBS), hydrocortisone, human fibroblast growth factor (hFGF), vascular endothelial growth factor (VEGF), human epidermal growth factor (hEGF), ascorbic acid, and antibiotics gentamicin and amphotericin. On the day of experiments, EGM2MV is switched for EBM, a serum-free basal media developed for normal human endothelial cells. EBM lacks FBS and the other growth factors found in EGM2MV to allow for minimal effect on experimental protocols.

### Determination of Endothelial Barrier Function

Barrier function of confluent HUVEC monolayers was determined by assessing transendothelial electrical resistance (TER), using an Electric Cell/Substrate Impedance Sensing (ECIS) ΖΘ system (Applied Biophysics, Troy, NY). ECIS provides a real-time, label-free, impedance-based method to study the barrier dynamics of cells grown in culture onto gold-filmed 8-well arrays containing surface electrodes and culture media serving as an electrolyte^54, 55^. Cells were subcultured onto gelatin-coated gold-plated 8W1E ECIS electrode arrays (1.5–1.u × 10^5^ cells/well) in EGM2MV medium. The arrays were attached to the ECIS station in a 37 °C, 5% CO_2_ incubator for overnight monitoring as the cells formed a confluent monolayer. Prior to experiments, the medium was changed to endothelial basal medium (EBM; Lonza) and the cells were allowed to reestablish a steady baseline TER within a range of 8,000–12,000 Ω. For ECIS experiments, cells were serum starved for at least 1–2 hours. HUVEC were used at passages 1–4 and only wells with displaying a steady baseline TER between 8,000–12,000 Ω were used for study. To confirm the effectiveness of S1P as an endothelial barrier enhancer at physiological concentrations, HUVEC were treated with 0.1, 0.2, 0.6, and 1.0 µM S1P. These concentrations fall within the ranges reported for plasma^21, 56^. To test the effectiveness of S1P in restoring TER to baseline levels following alcohol-induced endothelial barrier dysfunction, 1.0 µM S1P was added to HUVEC 5 minutes after 50 mM alcohol and compared with alcohol/vehicle cells.

### Data Analysis

All data are presented as means ± SE. In cases where comparisons between two groups were made, an unpaired Student’s t-test was used. For comparisons using the 2X2 design, which included sham versus hemorrhage, and water versus alcohol treatment, a two-way ANOVA was used. For comparisons between groups over time, a repeated-measures ANOVA design was utilized. When the initial AVOVA showed significance, post-hoc tests were used to compare intergroup differences. If there were only two groups to compare in this case, then Fisher’s LSD test was used. When the goal was to compare different groups to a control, Dunnett’s test was used. When the goal was to compare all groups to each other, Tukey’s test was used. Significance was accepted when P < 0.05. For the experiments utilizing rats, the sample sizes were determined using a sample size calculator with a 2X2 design or 4X1, a delta of 1.0, beta set at 0.8, and alpha at 0.05. For both the 2X2 and 4X1 designs, the minimum total number of rats needed was 24, or approximately 6 per group. These numbers were doubled assuming that only half of the experiments would reach completion, which was a required factor for inclusion in the final data. In most cases, sample sizes of the various treatment groups were kept roughly equal in order to minimize effects of unequal population variances that sometimes occur between the groups. Data analyses were performed using GraphPad Prism 6.0 software. Protocols and raw data will be made available upon request.
